# A novel technology for home monitoring of lupus nephritis that tracks the pathogenic urine biomarker ALCAM

**DOI:** 10.3389/fimmu.2022.1044743

**Published:** 2022-12-09

**Authors:** Rongwei Lei, Binh Vu, Katerina Kourentzi, Sanam Soomro, Adheesha N. Danthanarayana, Jakoah Brgoch, Suma Nadimpalli, Michelle Petri, Chandra Mohan, Richard C. Willson

**Affiliations:** ^1^ Department of Biomedical Engineering, University of Houston, Houston, TX, United States; ^2^ William A. Brookshire Department of Chemical and Biomolecular Engineering, University of Houston, Houston, TX, United States; ^3^ Department of Chemistry, University of Houston, Houston, TX, United States; ^4^ Division of Rheumatology, Johns Hopkins University School of Medicine, Baltimore, MD, United States; ^5^ Department of Biology and Biochemistry, University of Houston, Houston, TX, United States; ^6^ Escuela de Medicina y Ciencias de Salud, Tecnológico de Monterrey, Monterrey, NL, Mexico

**Keywords:** lupus nephritis, nanophosphors, lateral flow assay, biomarker, diagnostic

## Abstract

**Introduction:**

The gold standard for diagnosis of active lupus nephritis (ALN), a kidney biopsy, is invasive with attendant morbidity and cannot be serially repeated. Urinary ALCAM (uALCAM) has shown high diagnostic accuracy for renal pathology activity in ALN patients.

**Methods:**

Lateral flow assays (LFA) for assaying uALCAM were engineered using persistent luminescent nanoparticles, read by a smartphone. The stability and reproducibility of the assembled LFA strips and freeze-dried conjugated nanoparticles were verified, as was analyte specificity.

**Results:**

The LFA tests for both un-normalized uALCAM (AUC=0.93) and urine normalizer (HVEM)-normalized uALCAM (AUC=0.91) exhibited excellent accuracies in distinguishing ALN from healthy controls. The accuracies for distinguishing ALN from all other lupus patients were 0.86 and 0.74, respectively.

**Conclusion:**

Periodic monitoring of uALCAM using this easy-to-use LFA test by the patient at home could potentially accelerate early detection of renal involvement or disease flares in lupus patients, and hence reduce morbidity and mortality.

## Introduction

Systemic Lupus Erythematosus (SLE) is an autoimmune disease leading to chronic inflammation in multiple organs, including the kidneys (1). Renal involvement, termed lupus nephritis (LN), is a leading cause of morbidity and mortality (2). According to a new analysis funded by the Centers for Disease Control and Prevention (CDC), about 204,295 Americans have systemic lupus erythematosus (SLE) (3), based on strict criteria for diagnosis by the American College of Rheumatology. Parameters that raise suspicion for lupus nephritis include active urinary sediment with persistent hematuria, cellular casts, proteinuria, or elevated serum creatinine. The gold standard of diagnosis upon continued concern based on these parameters is a kidney biopsy. Once therapy has been initiated, similar parameters are used to determine if there is response to therapy. This includes reduction in proteinuria and urine sediment over a specified time frame based on treatment intervention. If goals are not met, then the recommendation is to alter the therapy and subsequently a repeat biopsy. However, the invasive nature, inter-observer variance, and attendant morbidity preclude frequent biopsies. It is noted that up to 60% of adults and 80% of children with SLE develop LN, with 10–30% progressing to ESRD within 15 years of diagnosis, despite aggressive treatment. Thus, early identification for prompt therapy can prevent progression to end stage renal disease and the need for transplantation or dialysis, and therefore improve patient quality of life (4–9).

The value of noninvasive biomarkers in categorizing disease activity of SLE and its renal involvement has been widely demonstrated. Comprehensive aptamer screening to identify lupus nephritis urinary biomarkers across ethnicities were carried out, where CD166 antigen (ALCAM) exhibited one of the highest discriminatory powers for active nephritis in African-Americans, Caucasians and Asians (10). The renal SLE Disease Activity Index (SLEDAI) is a score used to assess kidney disease activity and to reflect four kidney-related parameters including hematuria, pyuria, proteinuria, and urinary casts. The next-generation biomarker, urine ALCAM, distinguishes active LN (ALN) from non-active LN (quiescent or no prior nephritis), and previous LN, alluding to its role in renal SLE (11, 12). This was demonstrated in a study of 256 patients (ALN, active non-renal LN (ANR), inactive LN, inactive SLE) in a controlled cross-sectional study showing urine ALCAM (uALCAM) could be a strong biomarker for predicting renal histologic activity in LN and may serve as a valuable surrogate marker of renal histopathology (13). An investigation of uALCAM in 1038 patients with SLE/LN and controls from 5 ethnically-diverse cohorts demonstrated that uALCAM as a biomarker had distinguishing power irrespective of ethnicity (14). More importantly, in longitudinal modeling where a large multi-center cohort of LN subjects was studied, uALCAM correlated with changes in measures of disease severity, suggesting that the changes observed with uALCAM were due to the disease severity rather than nonspecific proteinuria changes (15). Finally, mechanistic studies in a preclinical model confirm that blocking the interaction of ALCAM with its ligand CD6 ameliorates LN, indicating that this biomarker is also a pathogenic disease driver (14). Thus, a large body of evidence supports the use of ALCAM as a diagnostic and disease monitoring biomarker for potential disease tracking of LN.

A point-of-care testing (POCT) platform’s importance rests on its potential to empower patients to monitor their health status with convenience, thus allowing for early diagnosis and monitoring of disease progression (16–19). The lateral flow assay (LFA) represents the most widely used rapid diagnostic POC testing platform. Briefly, antibody-conjugated reporters bind to the analyte in the clinical sample of interest. The analyte-conjugate complex flows along the nitrocellulose membrane on which specific antibodies are immobilized at predefined lines, where the analyte-conjugate complex forms a sandwich. Lastly, the absorbent pad absorbs any remaining sample of interest. For a given antibody, multiple reporters are available, but their detect-abilities vary. A recent review illustrates the detailed features of varied reporters for LFAs and their limitations (20).

Strontium aluminate doped with europium and dysprosium SrAl_2_O_4_:Eu^2^+,Dy^3^+ (SAO) is a persistent luminescent material resistant to photobleaching after extended exposure to 370 nm UV light (21). In this study, the assay reporters used were nanophosphor-based persistent luminescence nanoparticles (PLNPs) that emit intense visible light after excitation, lasting for hours. In previous research, both green-emitting SAO particles and blue-emitting [(Sr_0.625_Ba_0.375_)_1.96_Eu_0.01_Dy_0.03_] MgSi_2_O_7_ (SBMSO) particles (22) have been used to engineer LFAs for the detection of human chorionic gonadotropin (hCG) (21, 23), prostate-specific antigen (24), and herpes simplex virus type 2 antibodies (25), all imaged by smartphone (26). In this work, green-emitting SAO (rather than blue-emitting SBMSO) particles were used as reporters for biomarker detection given their higher brightness ratio, compatibility with urine samples, and better recovery after lyophilization.

In this study, we have set the foundation for building a portable at-home detection device to noninvasively assess lupus nephritis activity: a highly sensitive LFA coupled with smartphone-based time-gated imaging to detect urine CD166/ALCAM, a leading biomarker for LN. In this work, nanophosphor-based lateral flow immunoassays demonstrate promise in facilitating home-based smartphone-enabled monitoring of disease activity in LN. This may allow the proactive institution of therapeutics and even preventive strategies in LN, while minimizing treatment-related side effects.

## Materials and methods

### Initial LFA-based antibody screening

Several commercially-available monoclonal antibodies were evaluated in pairwise combinations both for capture and detection: eight anti-ALCAM antibodies and four anti-HVEM antibodies, shown in **Supplementary Tables S2**–**4**. Antibodies were conjugated to nanophosphors following our previously described protocol (24, 25). Antibodies provided with glycerol were buffer-exchanged in PBS pH 7.4 using Zeba Spin desalting columns (7K MWCO; Thermo Fisher Scientific). LFA strips were assembled using FF80HP nitrocellulose membrane (Cytiva) where test lines (TL) and control lines (CL) were manually spotted or dispensed using a BioDot dispenser (XYZ30600124) at a rate of 1 µl/cm. Two LFA running buffers (to facilitate the flow) were tested: running buffer-A1 (10 mM HEPES, 0.6% PVP40, 0.4% PEG (3350 ave. Mol. Wt, Sigma-Aldrich), 100 mM NaCl, 1% BSA, pH 7.25) and running buffer-H1 (1X PBS, 2% Tween-20, 25 mM NaCl, 0.5% NFDM, pH 8). Five µl of anti-ALCAM Ab- or anti-HVEM Ab-conjugated nanophosphors (5 µg) were mixed with 40 µl of corresponding running buffer spiked with 18 ng/ml of the analyte. 40 µl of the mixture was loaded to the LFA strip. Negative control was run for all antibody pairs. After 30 min, the strips were imaged on a FluorChem-based imaging platform (24, 25). Antibody pairs showing a high signal-to-noise ratio (SNR, calculated as the ratio of TL of the positive strip to the TL of the negative strip) and high TL/CL of the positive strip were selected for further LFA development.

### Functionalization of gold nanoparticles

AuNPs, 1 ml of OD =1 (40 nm, DCN Diagnostics #GC-020), were mixed with 100 µl of 4 mM KCl (J.T. Baker) in two low binding tubes. Then, 20 µl 0.5 mg/ml of mAb-2A or 10 µl 1mg/ml of mAb-1H were added for 30 min on a rotator at room temperature. Next, 100 µl of 10% BSA was added to block the Au nanoparticles. After 20 min on the rotator, the functionalized nanoparticles were collected by centrifugation (5 min, 10 000 rcf, room temperature). The particles were washed once in 1 ml of a storage solution (1% BSA and 10% sucrose (Sigma-Aldrich) in PBS pH 7.4), resuspended in 100 µl of storage solution and absorbance measured at 520 nm, and then stored at 4°C. LFA strips were assembled using the FF120HP nitrocellulose membrane (Cytiva). The test line was dispensed with 1 mg/ml anti-ALCAM (pAb-1A) or anti-HVEM (pAb-1H) capture antibody and the control line was dispensed with 0.5 mg/ml anti-mouse antibody at a rate of 1 µl/cm. Running buffer-A1 was used for both ALCAM and HVEM detection using AuNPs-based LFA and nanophosphor-based LFA. Five ul of AuNPs or nanophosphors were mixed with 40 µl of running buffer-A1 spiked to a serial dilution. 40 µl of the mixture was loaded onto the ALCAM or HVEM LFA strip.

### Lyophilization of functionalized nanophosphor reporters

To lyophilize nanophosphors, 3 µl of anti-ALCAM nanophosphors or anti-HVEM nanophosphors (120 µg) were mixed with 87 µl of Lyo buffer (4% trehalose, 4% mannitol (Sigma-Aldrich), 10 mM HEPES, 5% BSA, pH 7.25), in a low-binding tube and sonicated for 30 min. Then the tubes were transferred to -80°C freezer for 30 min and were then placed in an ice block and transferred to the lyophilization chamber (LabConco, -56°C, 0.090 Pa) overnight. The lyophilization chamber was ice precoated to maintain a low temperature before use. LFA strips were assembled using the FF120HP nitrocellulose membrane. The test line was dispensed with 1 mg/ml anti-ALCAM (pAb-1A) or anti-HVEM (pAb-1H) capture antibody and the control line was dispensed with 0.5 mg/ml goat anti-mouse antibody at a rate of 1 µl/cm. The lyophilized anti-ALCAM Ab or anti-HVEM Ab-conjugated nanophosphors test tube was added to 45 µl of water and incubated for 5 min. For ALCAM or HVEM detection, 5 µl of reconstituted nanophosphors were mixed with 40 µl of running buffer-II (10 mM HEPES, 0.7% PVP40, 0.3% Tween-20, 100 mM NaCl, 0.7% PEG, pH 7.25) spiked with analyte (ALCAM or HVEM) into a serial dilution. 40 µl of the mixture was loaded onto the ALCAM or HVEM LFA strip.

### Clinical sample testing using ALCAM-ELISA and HVEM-ELISA

The preliminary cross-sectional study included 107 urine samples from 30 renal active lupus nephritis (ALN), 18 active non-renal lupus (ANR), 29 inactive SLE patients, and 30 healthy controls (HC); all patients from Hopkins University School of Medicine, Baltimore, MD (**Supplementary Table S1**), and all HC from HC BioIVT (New York, USA). Samples were stored at -80°C. Patient disease activity, including SLEDAI and rSLEDAI, were evaluated as detailed before (27). The urine’s absolute ALCAM and HVEM concentrations were estimated using commercially available ELISA assays (Human HVEM/TNFRSF14 DuoSet ELISA, DY356 R&D Systems; Human ALCAM DuoSet ELISA, DY656, R&D Systems) and standard curves according to the manufacturer’s instructions. The 96-well ELISA plate was incubated with 50 µl 2 µg/ml of capture antibody in 1X PBS (pH 7.4) per well overnight at room temperature and washed three times with 200 µl of wash buffer (0.05% Tween-20, 1X PBS). The plate was then blocked with 150 µl of reagent diluent (1% BSA in 1X PBS, pH 7.4) per well for 1 h at room temperature and washed three times with wash buffer. Urine samples were 500-fold diluted in reagent diluent for HVEM validation and 25-fold dilution for ALCAM validation. 500 µl of diluted the sample was added to the pre-coated wells. After incubating the sample for 2 h at room temperature, the plate was washed three times with wash buffer, then 50 µl 0.1 µg/ml of detection antibody in reagent diluent was added to each well, followed by 2 h of incubation at room temperature. The plate was washed three times with wash buffer, followed by 50 µl of 1X streptavidin-HRP in reagent diluent per well for 20 min. The plate was washed three times with wash buffer, followed by 50 µl of TMB substrate per well for 20 min. Finally, 25 µl of stop solution (2 N H_2_SO_4_) was added. A microplate reader (ELX808, BioTek Instruments, Winooski, VT) was used to read the optical density at 450 nm. Urine creatinine concentrations were estimated using commercially available creatinine assays (KGE005 R&D Systems). Each well of the plate was loaded with 25 µl of urine diluted 20-fold in water, followed by 50 µl of 1 N NaOH: 0.13% Picric acid solution at a ratio of 1:5. After 30 min, the plate was read at 490 nm.

### Clinical sample testing by LFA

The urinary ALCAM and HVEM levels of the same 107 human samples were tested by separate LFAs using lyophilized anti-ALCAM conjugated nanophosphors and anti-HVEM conjugated nanophosphors, respectively. The test line was dispensed onto FF120HP nitrocellulose membrane with 1 mg/ml anti-ALCAM or anti-HVEM capture antibody, and the control line was dispensed with 0.25 mg/ml goat anti-mouse antibody at a rate of 1 µl/cm. Running buffer-II (10 mM HEPES, 0.7% PVP40, 0.3% Tween-20, 100 mM NaCl, 0.7% PEG (3350 ave. Mol. Wt, Sigma-Aldrich), pH 7.25) was used for both nanophosphors LFAs. 45 µl of water was added to the lyophilized nanophosphors and incubated for 5 min. For ALCAM detection, 20 µl of urine was mixed with 5 µl of reconstituted nanophosphors and 25 µl of running buffer-II and was applied to the ALCAM LFA strip. For HVEM detection, 5 µl of urine was mixed with 5 µl of reconstituted nanophosphors and 40 µl of running buffer-II and was applied to the HVEM LFA strip. After 35 min, the strips were imaged using a smartphone.

### Statistical analysis

Lateral flow assay (LFA) dose-response curves and biomarker data from LFA and ELISA were plotted and analyzed using either GraphPad Prism 5 (GraphPad, San Diego, CA) or R 3.4.1 (R Foundation for Statistical Computing, Vienna, Austria). Biomarker group comparisons by LFA and ELISA were analyzed using the Mann–Whitney U-test as datasets were not normally distributed, with statistical p-values as computed for each comparison. One-way ANOVA was used to analyze the LoD, linearity (r^2^), responsiveness, and average CV metrics among standard curves. The Pearson method was used for correlation analysis. Receiver Operating Curves (ROC) were used to assess and demonstrate the discriminative power of the biomarker as assayed by LFA and ELISA. Images from the FluorChem platform and smartphone were analyzed using ImageJ version 1.51 (100) (U. S. National Institutes of Health; Bethesda, MD). A macro (**Supplementary Methods #6**) was installed in ImageJ to set measurements (line width set to 8 pixels, scale to 1 pixel/unit ratio, removal of grid lines on plots, area integration, setting foreground/background to black). The brightness ratio was defined as the integrated brightness at the test line (TL) over the integrated brightness at the control line (CL). Standard curves of LFA performance were constructed relating the brightness ratio on LFA to the analyte concentration. The limit of detection (LoD) is the lowest concentration exceeding the sum of the mean of the blanks (n=2) and three times the standard deviation of the blank. The signal-to-noise ratio (SNR) of a given antibody pair was defined as the integrated brightness at the TL of a positive LFA strip with that antibody pair over the integrated brightness at the TL of a negative strip with the same antibodies but no analyte. R 3.4.1 was used to derive the SNR and brightness ratio of the designated positive strip (TL/CL) for selection of optimal antibody pairs.

Please refer to supplemental information for nanophosphors preparation, stabilization, functionality testing and strip assembly (**Supplementary Methods 1–4**). Further discussion regarding FluorChem-based imaging and smartphone-based imaging of nanophosphors is also available in **Supplementary Methods (#5)**.

## Results

In this paper, we focus on urine ALCAM detection as this biomarker has been extensively validated as a biomarker for lupus nephritis, as discussed above. Initial studies focused on identifying the optimal antibody pairs for detecting uALCAM by LFA and designing/optimizing the LFA, while the later studies focused on assessing the diagnostic potential of uALCAM LFA in LN.

### Antibody pair selection and construction of LFA strips for assaying ALCAM

**Figure 1:**
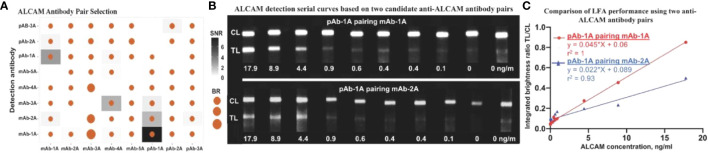
Antibody pair selection for construction of ALCAM LFA. **(A)** 57 antibody pairs (eight types of antibodies were tested in all combinations and with each serving as detection Ab-the antibody conjugated onto the nanophosphors and as capture Ab-the antibody immobilized onto the LFA strip) were tested for ALCAM LFA performance, of which two pairs showed relatively high SNR and brightness ratio. **(B)** Serial curve of nanophosphor-based LFA were constructed for ALCAM detection in buffer. Each concentration point was singularly tested except for negative controls (0 ng/ml) in duplicate. These grayscale images were collected using a FluorChem gel documentation and were analyzed using ImageJ. **(C)** Integrated brightness ratio (TL/CL) as a function of ALCAM concentration showed that pAb-1A as capture antibody and mAb-1A as detection antibody achieved the highest LFA performance with high linearity (r^2^) of 1. The positive (17.9 ng/ml) and negative (0 ng/ml) buffer under both candidate antibody pairs were tested twice before constructing the standard curves. SNR, signal-to-noise ratio (In a given antibody pair, the integrated brightness at the TL of that positive LFA strip over the integrated brightness at the TL of that negative strip with no analyte).; BR, Brightness ratio (the integrated brightness intensity of TL over that of CL); TL, test line; C,: control line; pAb-1A, polyclonal anti-ALCAM antibody from R&D; mAb-1A, monoclonal anti-ALCAM antibody from R&D; The other antibody types tested were purchased from Biolegend, Thermo Fisher Scientific, Santa Cruz, and ABclonal, as detailed in **Supplementary Tables S2**–**4**.

57 antibody pairs were assessed in order to identify pairs with high SNR and TL/CL. Besides SNR and brightness ratio, lower aggregation of functionalized nanoparticles was also considered in choosing the type of antibody to conjugate onto nanoparticles. In total, 57 antibody pairs (eight types of antibodies) were tested for anti-ALCAM antibody pair selection (**Figure 1A**), of which two pairs (capture antibody pAb-1A paired with detection antibody mAb-1A and capture antibody pAB-1A paired with detection antibody mAb-2A) showed relatively high SNR and brightness ratio. Standard curves of ALCAM detection by nanophosphor LFA based on these two pairs are shown in **Figure 1B**. The correlation between ALCAM concentration and integrated brightness ratio (TL/CL) is shown in **Figure 1C**. Notably, pAb-1A as capture antibody paired with mAb-1A as detection antibody achieved the highest sandwich immunoassay performance with a high linearity (r^2^) of 1 (compared to 0.93 for the other leading antibody pair) and an overall higher integrated brightness ratio. Thus, this configuration was chosen as detection antibody for further ALCAM detection assays.

### Comparison of gold- and nanophosphor-based ALCAM LFAs

**Figure 2:**
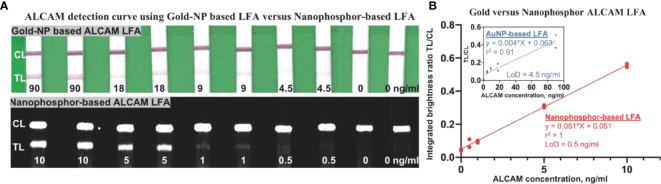
Comparison of ALCAM detection by nanophosphor- and AuNP-based LFA. **(A)** Scanned images of LFAs of dilution series of ALCAM using AuNPs and nanophosphors. The AuNPs-based LFA used pAb-1A as capture antibody and mAb-2A as detection antibody, and the SAOs-based LFA used pAb-1A as capture antibody and mAb-1A as detection antibody. **(B)** The correlation between ALCAM concentration and integrated brightness ratio as assayed using AuNP-LFA (inside box) and nanophosphor-based LFA. pAb-1A, polyclonal anti-ALCAM antibody from R&D; mAb-1A, monoclonal anti-ALCAM antibody from R&D; mAb-2A, monoclonal anti-ALCAM antibody from Bio-legend; detection Ab, the antibody conjugated onto the SAOs; capture Ab, the antibody immobilized onto the LFA strip; LoD, limit of detection, the lowest concentration exceeding the sum of the mean of the blanks (n=2) plus three times the standard deviation of the blanks.

To compare the performance of nanophosphor-based LFA in ALCAM detection to traditional colorimetric colloidal gold nanoparticles (AuNPs), pAb-1A as capture antibody and mAb-1A as detection antibody were utilized for nanophosphor-based LFA, while mAb-2A as detection antibody was utilized for AuNP-based LFA. This selection was based on the high background of pAb-1A paired with mAb1-A in AuNP-based LFA (data not shown). Nanophosphors and AuNPs were functionalized separately, and 40 µl of running buffer-A1 was used to drive the flow of AuNPs and nanophosphors in the experiments shown in **Figure 2A**. For both LFAs, ALCAM detection standard curves were constructed. **Figure 2B** demonstrates the correlation between ALCAM concentration and integrated brightness ratio using the AuNP- and nanophosphor-based LFA. With nanophosphor-based LFA, the r^2^ and LoD of ALCAM detection were 1 and 0.5 ng/ml, respectively. With AuNPs-based LFA, the r^2^ and LoD of ALCAM detection were 0.91 and 4.5 ng/ml, respectively. Thus, ALCAM detection using a nanophosphor-based LFA outperformed traditional AuNPs-based LFA in linearity (r^2^) and limit of detection, and were used for the rest of the assays.

### ALCAM LFA validation

**Figure 3:**
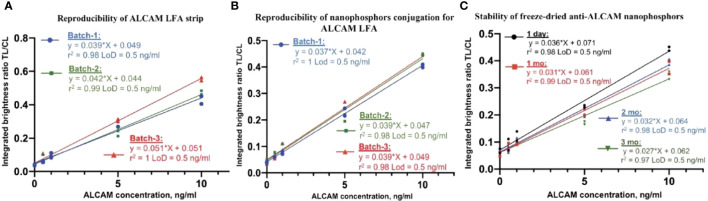
ALCAM LFA assay validation. **(A)** Three different batches of anti-ALCAM (pAb-1A as capture antibody) dispensed strips were assembled. One batch of anti-ALCAM (mAb-1A as detection antibody) conjugated nanophosphors was mixed with spiked running buffer-1A and loaded onto ALCAM LFA strips from three batches. No significant differences were found in linearity and LoD (0.5 ng/ml) among the three standard curves. **(B)** Assessing the reproducibility of anti-ALCAM nanophosphors preparation. Three batches of conjugated anti-ALCAM nanophosphors were individually mixed with spiked running buffer-1A and loaded onto the same batch of ALCAM LFA strips. No significant differences were found in linearity and LoD (0.5 ng/ml) among the three batches of conjugated anti-ALCAM nanophosphors. **(C)** Stability of anti-ALCAM conjugated nanophosphors. Anti-ALCAM conjugated nanophosphors were freeze-dried and then reconstituted after one day, one month, two months, and three months after storage, respectively. No significant differences were found in linearity and LoD (0.5 ng/ml) after different storage durations, verifying the stability of lyophilized nanophosphors at room temperature.

To assess the reproducibility of ALCAM LFA strip preparation, three different batches of anti-ALCAM strips (pAb-1A as capture antibody) were assembled and stored with desiccant at room temperature. To verify the reproducibility of the anti-ALCAM (mAb-1A as detection antibody) conjugation process, three different batches of anti-ALCAM conjugated nanophosphors were prepared and tested. The reproducibility of ALCAM LFA strip preparation was verified by showing no significant difference in the linearity r^2^ and LoD (0.5 ng/ml) among the three batches by one-way ANOVA (**Figure 3A**). Likewise, the reproducibility of anti-ALCAM conjugated nanophosphors preparation was also confirmed (**Figure 3B**).

Stabilizers such as sucrose, trehalose, mannitol, dextran, BSA, glycine, Tween-20, and PVP40 (28) were tested for their protective effect (data not shown). Notably, freeze-dried nanophosphors in the presence of 1:1 mannitol and trehalose produced cakes with excellent integrity and ease of reconstitution compared to the collapsed cakes observed with trehalose alone (data not shown). In this research, a solution of 4% trehalose and 4% mannitol was used For ease of transportation and storage, conjugated nanophosphors were freeze-dried in Lyo buffer. The anti-ALCAM nanophosphors’ stability was assessed by using ALCAM LFA after storage at room temperature for one day, one month, two months, and three months, as shown in **Figure 3C**. At each time point, the reconstituted nanophosphors at different ages were mixed with running buffer-II and loaded onto ALCAM LFA strips from the same batch (stored with desiccant at room temperature) to construct serial curves. The stability of lyophilized anti-ALCAM nanophosphors was verified by demonstrating no significant differences between the linearity r^2^ and LoD (0.5 ng/ml) values of the respective standard curves, using one-way ANOVA.

### Comparing uALCAM detection in clinical samples by LFA and ELISA

**Figure 4:**
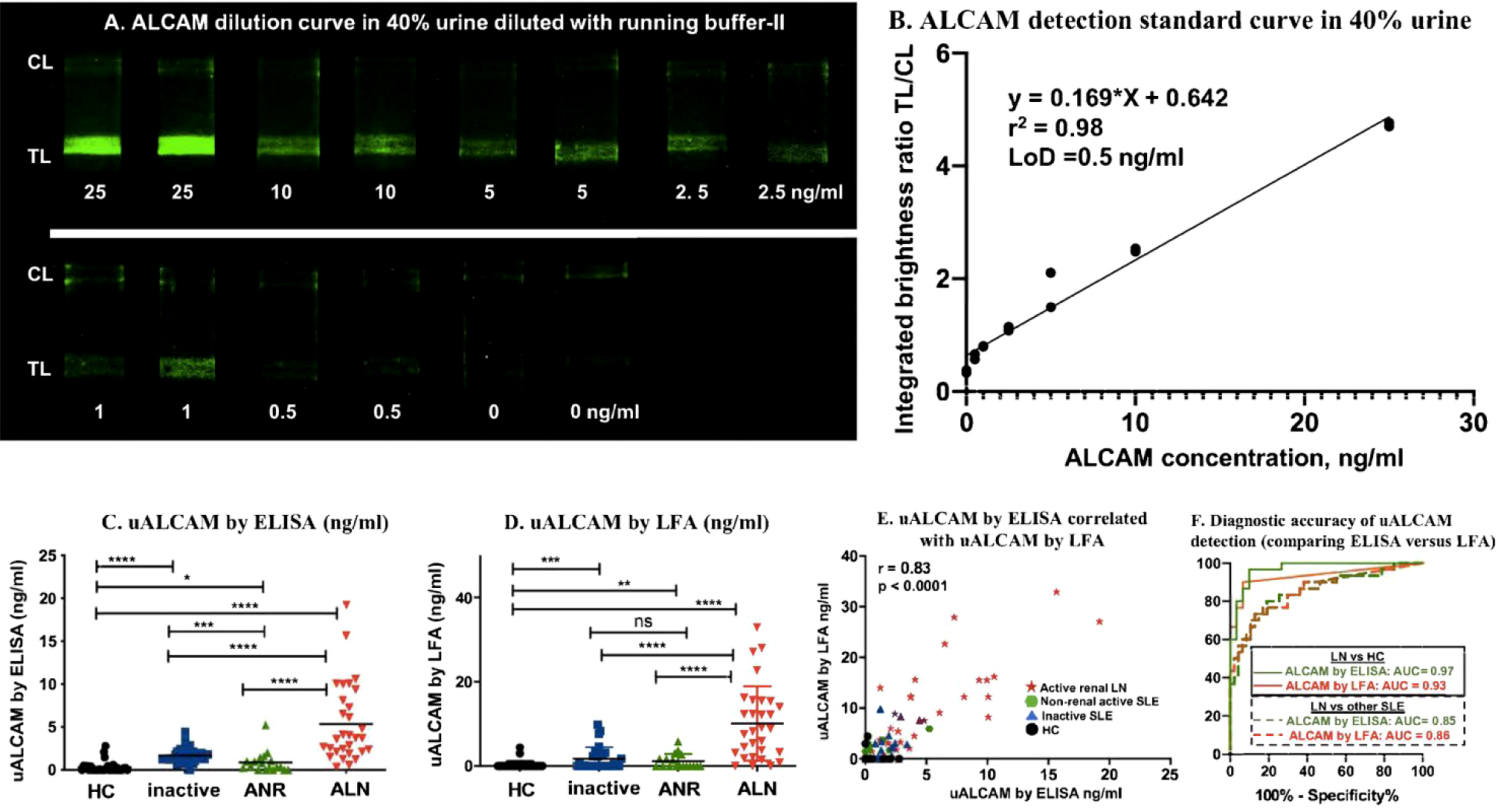
uALCAM detection by LFA and ELISA in clinical samples. **(A)** Samples from ALCAM standard curve constructed in 40% urine were run on ALCAM LFA using lyophilized anti-ALCAM nanophosphors. **(B)** The ALCAM standard curve LFA strips were imaged using a smartphone, and the correlation of ALCAM concentration with TL/CL ratios is shown. This regression equation was then used to convert the LFA TL/CL ratios observed in the clinical samples to ALCAM concentration. The column graphs showed the uALCAM levels in four groups of samples (30 HC, 29 inactive, 18 ANR, and 30 ALN) as assayed by ELISA **(C)** and LFA **(D)** The plots show the mean concentration in urine for each disease group. *p < 0.05, **p < 0.01, ***p < 0.001, ****p < 0.0001 as determined using Mann–Whitney U-test. **(E)** Plotted is the Pearson correlation of uALCAM assayed by ELISA versus uALCAM assayed by LFA. **(F)** uALCAM as assayed using LFAs had the capacity to distinguish ALN patients from healthy subjects with high accuracy values (ROC AUC=0.93, solid red line) and from all other lupus patients (ROC AUC=0.86, dashed red line), and were very comparable to the performance metrics of the conventional ELISA assay. HC, healthy control; inactive, inactive SLE patients; ANR, active non-renal SLE patients; ALN, active renal lupus nephritis patients.

To compare the anti-ALCAM LFA against the conventional ELISA counterpart, 107 urine samples were measured by nanophosphor ALCAM LFA and by commercial ALCAM ELISA. Standard curves of ALCAM in 40% urine diluted with running buffer-II were constructed using ALCM standards of 0, 0.5, 1, 2.5, 5, 10, 25 ng/ml, each measured in duplicate (**Figure 4A**). The urine sample used to construct the serial dilution was a healthy control with ALCAM concentration below the LoD of the ELISA (62.5 pg/ml). The correlation between ALCAM concentration and TL/CL is plotted in **Figure 4B**, and this was used to convert the ALCAM LFA TL/CL results to concentration units. Four groups of samples including healthy control (HC), inactive lupus (inactive), active non-renal lupus (ANR), active lupus nephritis (ALN) were compared. Urinary ALCAM (uALCAM) assayed both by ELISA (**Figure 4C**) and by LFA (**Figure 4D**) was able to distinguish the ALN patients from the other groups. A high Pearson correlation was observed between the two assays (r =0.83, p < 0.0001) (**Figure 4E**). Importantly, urine ALCAM LFA had the capacity to distinguish ALN patients from healthy subjects with high accuracy (ROC AUC=0.93, solid red line) and from all other lupus patients (ROC AUC=0.86, dashed red line), and were very comparable to the performance of the conventional ELISA assay (**Figure 4F**). Importantly, uALCAM as assayed by LFA, correlated strongly with disease activity measurements, including SLEDAI and rSLEDAI (Spearman correlation of r=0.50, p<0.0001, and r = 0.65, p<0.0001, respectively; **Supplementary Figures 2A, B**).

### Antibody pair selection for construction of HVEM LFA and its characterization in nanophosphor-and AuNP-based LFA

To correct for variations in individual hydration status, urinary creatinine (Cr) is routinely used to normalize urine biomarker levels. However, the small molecular weight of Cr (113 Da) makes the direct sandwich lateral flow immunoassay difficult and well-validated anti-Cr antibodies are lacking at the time of this report, limiting their potential use in sandwich antibody-based point of care applications. A recent aptamer-based screening of urine 1129 proteins has reported other potential protein alternatives to Cr, including HVEM, which was significantly correlated with urinary Cr in both Caucasian (Pearson r = 0.72, p < 0.0001) and African-American subjects (Pearson r = 0.7, p = <0.0001) (27). In order to use urine HVEM instead of urine Cr for normalizing urine biomarker levels, we next designed and optimized the detection of urinary HVEM (uHVEM) using the LFA test format.

**Figure 5:**
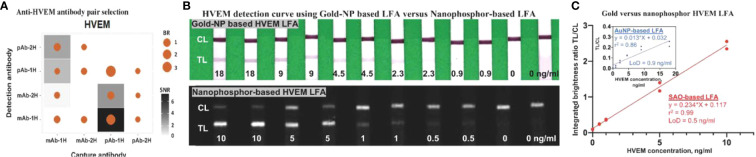
Antibody pair selection for construction of HVEM LFA and its characterization in nanophosphor-and AuNP-based LFA. **(A)** 13 antibody pairs (four types of antibodies) were tested for HVEM LFA performance, of which one pair (capture antibody pAb-1H paired with detection antibody mAb-1H) showed the highest SNR and BR. **(B)** Scanned images of the standard curve of HVEM as assayed using AuNPs-based and nanophosphor-based LFA, and pAb-1H as capture antibody and mAb-1H as detection antibody. **(C)** Correlation between HVEM concentration and integrated brightness ratio as assayed using AuNPs-LFA (inside box) and nanophosphor-based LFA. pAb-1H: polyclonal anti-HVEM antibody from R&D; mAb-1H: monoclonal anti-HVEM antibody from R&D; detection Ab: the antibody conjugated onto the nanophosphors; capture Ab: the antibody immobilized onto the LFA strip; LoD, limit of detection, the lowest concentration exceeding the sum of the mean of the blanks (n=2) plus three times the standard deviation of the blanks.

Thirteen pairs of anti-HVEM antibodies were assessed in order to identify pairs with high SNR and TL/CL. Besides SNR and brightness ratio, lower aggregation of functionalized nanoparticles was also considered in choosing the type of antibody to conjugate onto nanoparticles. In total, 13 antibody pairs (four types of antibodies) were tested for anti-HVEM antibody pair selection (**Figure 5A**), of which capture antibody pAb-1H paired with detection antibody mAb-1H showed the highest SNR and brightness ratio. To compare the performance of nanophosphor-based LFA in HVEM detection to traditional colorimetric colloidal gold nanoparticles (AuNPs), pAb-1H as capture antibody and mAb-1H as detection antibody were utilized for nanophosphor-based LFA and AuNPs-based LFA. Nanophosphors and AuNPs were functionalized separately, and 40 µl running buffer-A1 was used to help the flow of AuNPs and nanophosphors. For both LFAs, standard curves were constructed. **Figure 5B** demonstrates the correlation between HVEM concentration and integrated brightness ratio using the AuNP- and nanophosphor-based LFA. With nanophosphor-based LFA, the r^2^ and LoD of HVEM detection were 0.99 and 0.5 ng/ml, respectively. With AuNP-based LFA, the r^2^ and LoD of HVEM detection were 0.86 and 0.9 ng/ml, respectively. Thus, HVEM detection using a nanophosphor-based LFA outperformed traditional AuNPs-based LFA in linearity (r^2^) and limit of detection, and were hence used for the rest of the assays.

The reproducibility of HVEM LFA strip preparation, and the anti-HVEM (mAb-1H as detection antibody) conjugation process, and the stability of freeze-dried anti-HVEM nanophosphors at room temperature were separately verified (**Supplementary Figure S1**), demonstrating no significant difference in linearity r^2^ and LoD (0.5 ng/ml) among the different conditions.

### Comparing uHVEM detection using LFA and ELISA in clinical samples

**Figure 6:**
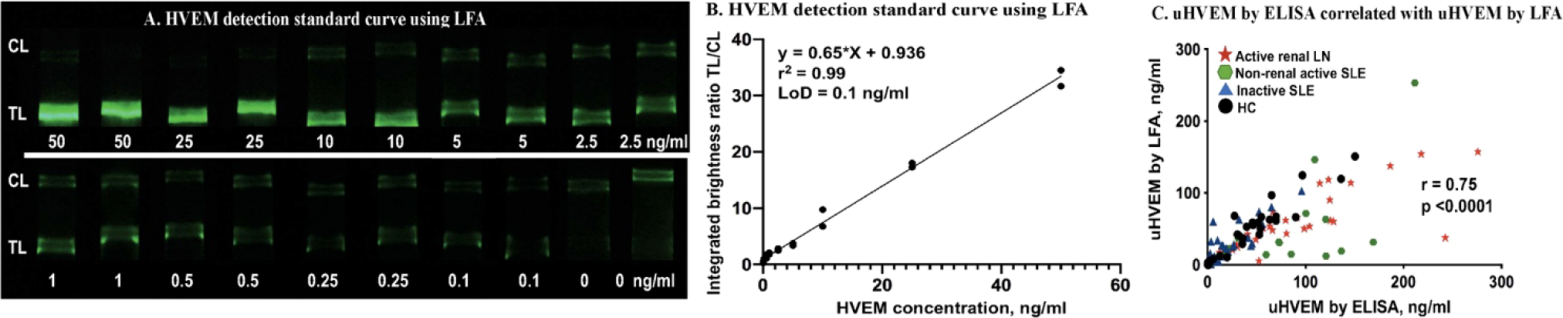
uHVEM detection by LFA and ELISA in clinical samples. **(A)** An HVEM standard curve in buffer was constructed using lyophilized anti-HVEM nanophosphors. **(B)** The HVEM standard curve LFA strips were imaged using a smartphone, and the correlation of HVEM concentration with TL/CL ratios is shown. This regression equation was then used to convert the LFA TL/CL ratios observed in the clinical samples to HVEM concentration. **(C)** Plotted is the Pearson correlation of uHVEM assayed by ELISA versus uHVEM assayed by LFA.

To compare the anti-HVEM LFA against its conventional ELISA counterpart, the same 107 urine samples (healthy control (n=30), inactive lupus (n=29), active non-renal lupus (n=18), active lupus nephritis (n=30)) were measured by newly-fabricated HVEM LFA strips and by commercial HVEM ELISA. Standard curves of HVEM detection in buffer were constructed using HVEM standard (0, 0.1, 0.25, 0.5, 1, 2.5, 5, 10, 25, 50 ng/ml), each measured in duplicates (**Figure 6A**). The correlation between HVEM concentration and TL/CL is plotted in **Figure 6B**, and this was used to convert the HVEM LFA TL/CL results to concentration units. A high Pearson correlation was observed between these two assays (r =0.75, p < 0.0001) (**Figure 6C**).

### Cross-reactivity and strip stability profiles of ALCAM LFA and HVEM LFA

Basic Fibroblast growth factor (bFGF, Cat: DY233), Tumor Necrosis Factor-alpha (TNF-α, Cat: DY210), Interleukin-6 (IL-6, Cat: DY206), Cystatin c (Cat: DY1196), and Connective tissue growth factor (CTGF, Cat: DY9190-05) were all purchased from R&D system and tested as interferents at concentrations within or much higher than the physiological range in patients (29–33). Absolute urinary interferent concentration that was reported as a normalized level by uCr was estimated throughout uCr 1 mg/ml. Urinary bFGF was reported to reach a maximum uCr-normalized level of 4.9 pg/µg (ubFGF at 4.9 ng/ml), in juvenile pilocytic astrocytoma (29) and was tested as an interferent at 150 ng/ml (30 times the maximum); urinary TNF-α was reported to reach a maximum uCr-normalized level of 16.3 ng/g (uTNF-α at 16.3 pg/ml) in acute interstitial nephritis (30) and was tested as an interferent at 130 pg/ml (9 times the maximum); urinary IL-6 was reported with a uCr-normalized cutoff level of 75 pg/mg (uIL-6 at 75 pg/ml) in acute kidney injury (31) and was tested as an interferent at 120 pg/ml (1.6 times the cutoff); urinary cystatin c (32) was reported with a cutoff level of 120 ng/ml in acute kidney injury and was tested as interferent at 125 ng/ml; CTGF was reported with a uCr-normalized maximum level of 470 pmol/g (uCTGF at 17.9 ng/ml based on 38 kDa molecular weight) in interstitial fibrosis and tubular atrophy after renal transplantation (33). CTGF was tested as interferent at 100 ng/ml (5.6 times the maximum).

**Figure 7:**
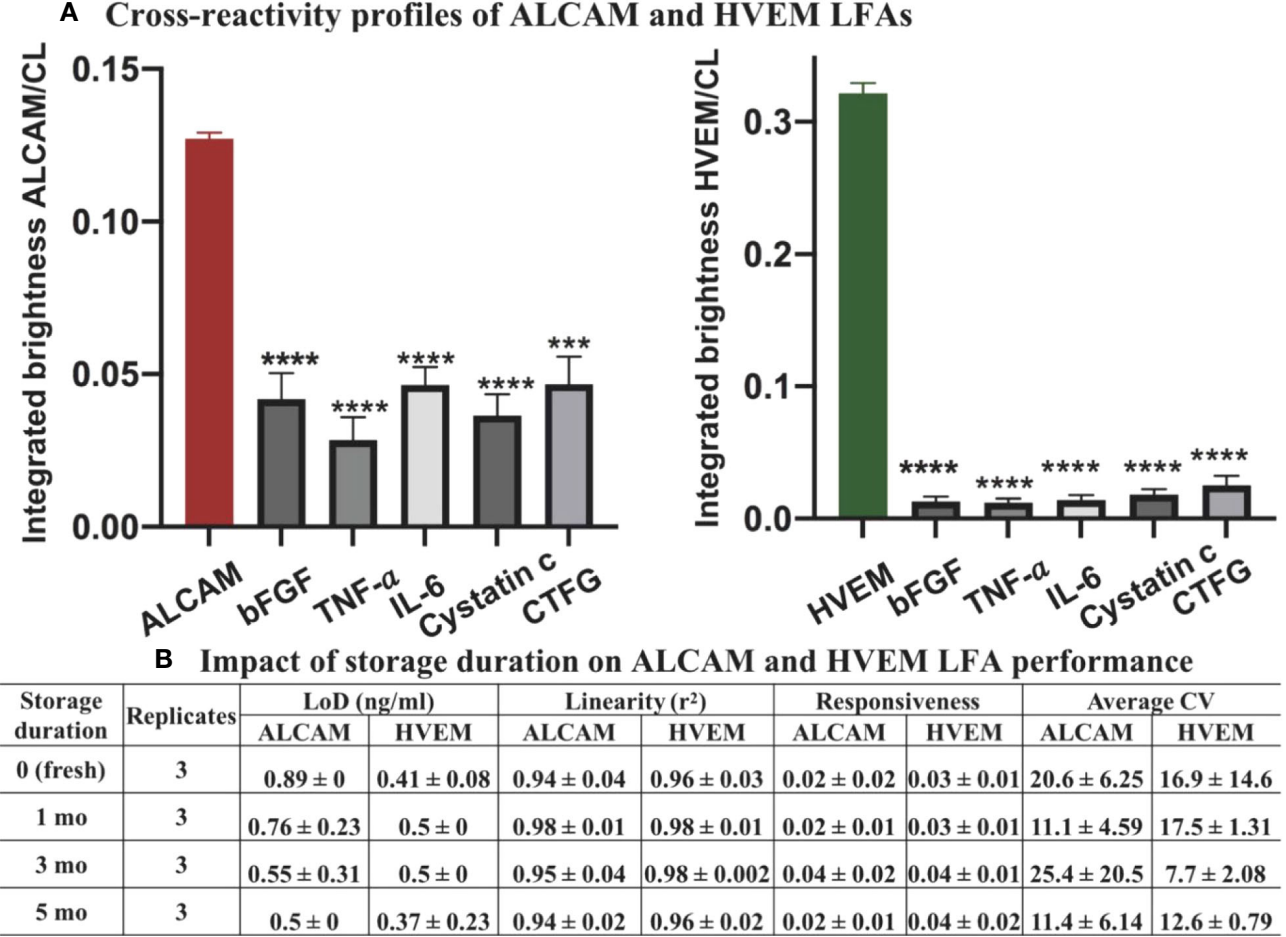
Cross-reactivity and LFA strip stability profiles of ALCAM LFA and HVEM LFA. **(A)** 1.5 ng/ml of ALCAM or HVEM (positive controls) or bFGF (150 ng/ml), TNF-α (130 pg/ml), IL-6 (120 pg/ml), cystatin c (125 ng/ml), and CTGF (100 ng/ml) were individually spiked to running buffer and assayed using the LFAs. Samples were loaded onto the ALCAM LFA (left) or HVEM LFA (right) in triplicates. ***p < 0.001, ****p < 0.0001 compared to the positive control, as determined using unpaired t-test. **(B)** The impact of storage duration on strip performance was next tested at room temperature over five months, in triplicate. Three repeats of standard curves were constructed at each storage timepoint. No significant differences (one-way ANOVA) were observed among the groups in terms of LoD, responsiveness, linearity, and average CV. Linearity (r^2^): goodness-of-fit measure for linear regression model; responsiveness: the slope of the regression model (signal=slope*concentration + intercept); average CV: average of the coefficient of variance across three standard curves at each timepoint.

Considering the uALCAM in LN patients is significantly greater than 1 ng/ml, the positive control samples was 1.5 ng/ml ALCAM spiked in running buffer-A, which is three times the limit of detection of ALCAM. Considering the uHVEM across patients and healthy controls is significantly greater than 1 ng/ml, the positive control sample was also 1.5 ng/ml HVEM spiked in running buffer-A, fifteen times the limit of detection of HVEM. To assess the specificity and cross-reactivity profiles of the ALCAM LFA and HVEM LFA tests, ALCAM or HVEM (which should yield positive results in the respective LFAs) and various other LN irrelevant proteins that may be present in kidney injuries and cancer relevant proteins, were tested for their signals when applied to these LFAs (which should yield significantly lower signal than positive strip) in **Figure 7A**.

Following Method 3.2 (nanophosphor-based LFA procedures), the ALCAM LFA and HVEM LFA were run in triplicate. The impact of each interferents was considered negligible for ALCAM or HVEM LFAs since the integrated brightness of all negative samples (interferents spiked) was significantly lower than that of the positive sample (no interferent-spiked) (**Figure 7A**). The results indicate that the developed LFA test strips are highly specific for ALCAM or HVEM detection, respectively.

To evaluate the stability of the ALCAM and HVEM LFA at room temperature, the prepared ALCAM LFA strips and HVEM LFA strips were individually stored at room temperature in 50 ml conical screw cap centrifuge tubes along with a silica gel desiccant for different durations - one day (fresh), one month, three months, or five months. No significant differences were observed among the groups in terms of LoD, responsiveness, linearity, and average CV (**Figure 7B**).

### Normalized uALCAM levels in clinical samples

**Figure 8:**
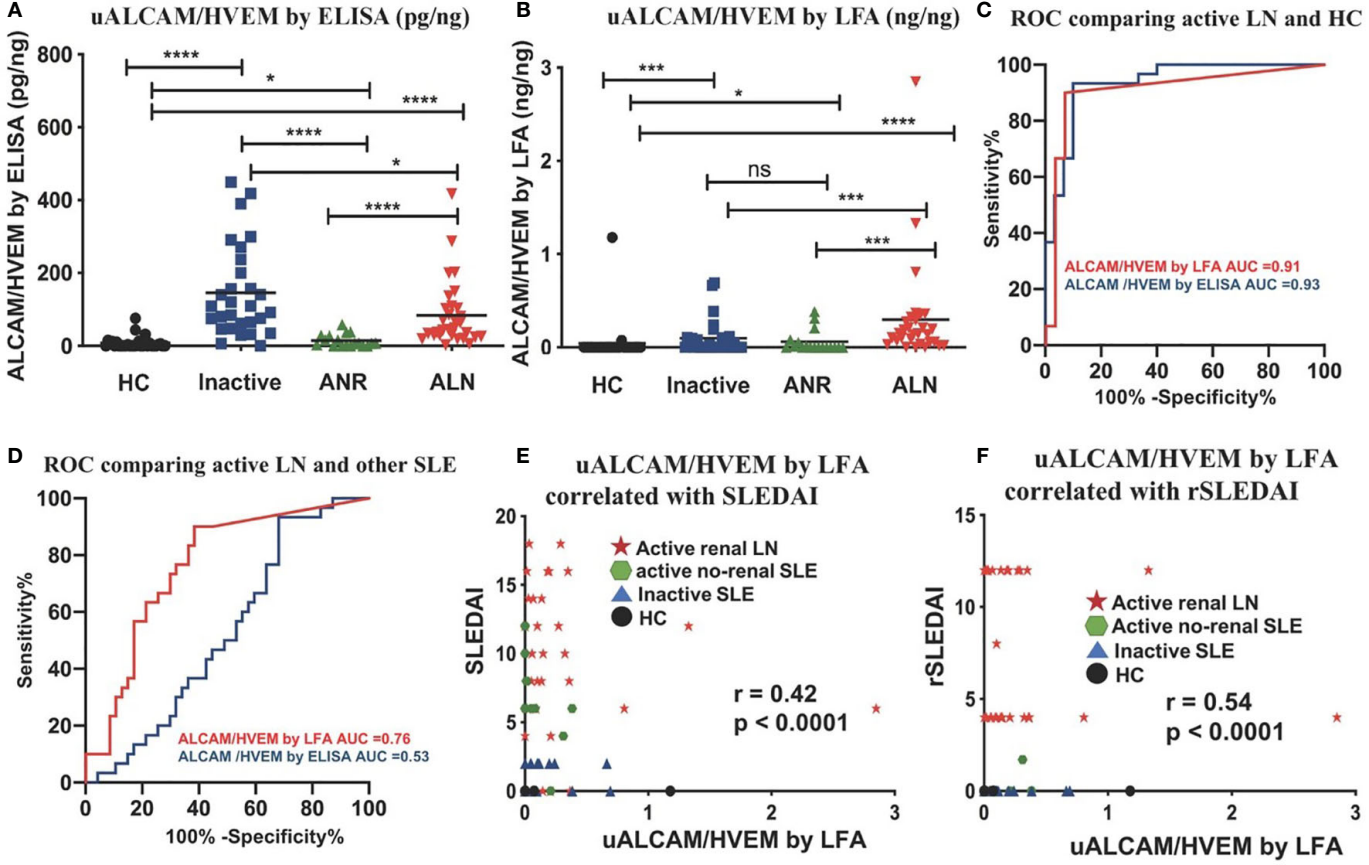
Comparing normalized uALCAM detection using ELISA and LFA in clinical samples. The column graphs show the uHVEM normalized uALCAM levels in four groups of subjects (30 HC, 29 inactive, 18 ANR, and 30 ALN) as assayed by ELISA **(A)** and LFA **(B)** The plots show the mean concentration in urine for each disease group. *p < 0.05, ***p < 0.001, ****p < 0.0001 as determined using Mann–Whitney U-test. **(C, D)** HVEM normalized uALCAM as assayed using the LFAs had the capability to distinguish ALN patients from healthy subjects with high accuracy values (ROC AUC = 0.91) and from all other lupus patients (ROC AUC = 0.76), and were significantly (Spearman) correlated with the disease activity metrics, SLEDAI **(E)** and rSLEDAI **(F)**. HC, healthy control; inactive, inactive SLE patients; ANR, active non-renal SLE patients; ALN, active renal lupus nephritis patients; (r)SLEDAI, (renal) SLE Disease Activity Index (SLEDAI).

We initially evaluated the test accuracy of the ALCAM LFA without normalizing the urine ALCAM levels against Cr (or any other marker; **Figure 4**). However, to assess the diagnostic metrics of urine biomarkers, they are usually normalized to urine Cr, to correct for hydration status. Since it is not readily feasible to incorporate Cr into the LFA test format, here we used urine HVEM as an alternative to urine Cr, based on previous reports indicating that urine HVEM and urine Cr are correlated (27). uALCAM and uHVEM levels were measured in 107 urine samples using the conventional ELISA and newly fabricated LFAs, to derive uHVEM- normalized uALCAM levels, as determined by ELISA (**Figure 8A**) and by LFA (**Figure 8B**).

The normalized uALCAM assayed both by ELISA and by LFA exhibited a high capacity to distinguish ALN patients from healthy subjects with high accuracy values (ROC AUC = 0.93 by ELISA; ROC AUC = 0.91 by LFA) (**Figure 8C**). Surprisingly, in this cohort, ELISA-derived HVEM-normalized uALCAM assayed by ELISA lost the ability to distinguish ALN patients from all other lupus patients (ROC AUC = 0.53), while LFA-derived HVEM-normalized uALCAM assayed by LFA had a high capacity to distinguish ALN patients from all other lupus patients (ROC AUC = 0.76) (**Figure 8D**). The disease activity measures, SLEDAI (**Figure 8E**) and rSLEDAI (**Figure 8F**) from all 107 subjects were collected and analyzed against LFA-derived HVEM-normalized uALCAM assayed by LFA, showing a good Spearman correlation of r=0.42, p<0.0001, and r = 0.54, p<0.0001, respectively. Importantly, ELISA-derived HVEM-normalized uALCAM assayed by ELISA was significantly correlated with LFA-derived HVEM-normalized uALCAM assayed LFA (r=0.53, p<0.0001, **Supplementary Figure 2C**).

## Discussion

Renal damage secondary to lupus nephritis remains prevalent in the patient population thus requiring early diagnosis and aggressive treatment. The gold standard for diagnosis of LN is a renal biopsy, allowing for characterization into classes, as well as measurement of activity and chronicity, which guide current treatment approaches. The goal of therapy is to avoid progression to advanced sclerosis or class VI LN which involves greater than 90% of glomeruli, leading to end-stage renal disease (ESRD), necessitating transplantation or dialysis. After initiation of therapy, response is assessed and if clinical goals are not met, a repeat biopsy is pursued. Unfortunately, frequent renal biopsies are not feasible because of their invasive nature and attendant morbidity (1–9). The biological fluid likely to reflect ongoing pathology in the kidney most closely is urine, which is also very compatible with POC- or self-testing. Hence, there is a clear need for an easy-to-use and accurate POC test for monitoring renal disease activity in lupus.

The CD166 antigen (ALCAM) exhibits one of the highest discriminatory powers for active lupus nephritis in the African-American, Caucasian, and Asian populations. As a next-generation biomarker, urine ALCAM distinguishes active LN from never LN (quiescent or no prior nephritis), previous LN, and controls with high accuracy (10, 11, 13, 14). Indeed, by following study protocol (**Supplementary Figure 3**), we have further confirmed the diagnostic potential of uALCAM for active LN. As assayed using the uALCAM LFA test, this biomarker distinguishes active LN patients from all other lupus patients with an accuracy value of 0.86, being comparable to that of uALCAM assayed by ELISA, with strong correlation with global and renal disease activity. Surprisingly, the performance of uALCAM (assayed by LFA) without any normalization for hydration status outperformed HVEM normalized uALCAM (assayed by LFA) with improved accuracy values for identifying active LN (0.93 vs 0.91) from HC, and for identifying active LN (0.86 vs 0.76) from all other lupus patients. Although these findings need to be validated in larger, independent cohorts, they raise hope that the assaying of a single analyte (without having to assay a normalizing protein or Cr) may meet the clinical diagnostic needs at the point of care. This greatly simplifies assay design and final cost to the patient.

On the other hand, several studies also suggest that multi-marker panels may exhibit improved measurement of LN activity, though no multi-marker panels have yet been independently reproduced across multiple independent test sites. Of importance, a couple of other urine proteins have also been independently validated as having excellent correlation with LN activity. Studies showed urine sCD163 discriminated patients with active LN from other SLE patients and was significantly elevated in proliferative LN. It strongly correlated with concurrent disease activity index and several specific pathological attributes, demonstrating its potential in predicting renal pathology (34–37). VCAM-1 is another example of a urinary biomarker that has been extensively investigated in LN, being predictive of disease activity and long-term renal function deterioration (10, 11, 38–41). A more recent report documents Interkukin-16-producing cells at key sites of kidney injury, implicating IL-16 in LN pathogenesis, designating it as a potentially treatable target and biomarker (42). These are just some examples of urinary biomarkers that could be multiplexed for diagnostic use once there is a compelling body of evidence demonstrating that the combination panel offers improved diagnostic capability compared to the single analytes.

The lateral flow assay (LFA) represents the most widely used rapid diagnostic POC testing platform, most commonly as the urine pregnancy test strip. The lyophilized antibody conjugated nanophosphors are first reconstituted in water in this LFA architecture and then mixed with a clinical sample of interest. The analyte-conjugate complex flows through the nitrocellulose membrane, where specific antibodies are immobilized at predefined lines, where the analyte-conjugate complex forms a sandwich. When coupled with smartphone flash as an energy source, the nanophosphors on the test line and control line emit green light which is captured by the phone. The nanophosphor-based LFA reported here demonstrates high reproducibility in strip preparation and particle conjugation and exhibits high stability in strip and lyophilized particle storage at room temperature. To the best of our knowledge, no published research has assessed the usability of conjugated nanophosphors at the level of assay validation reported in this work, although Clip health has obtained FDA EUA for an LFA using nanophosphors for qualitative rapid SARS-CoV-2 detection. This work also represents the first report of a platform for monitoring urine biomarkers using a smartphone to capture signals released by nanophosphors.

The ease of assay performance of an LFA test, affordable cost, rapid availability of test results, quantitative assay readout, coupled with the need for minimal equipment (only a cell phone in this case) render this platform compatible with home testing (by the patient) or rapid testing at the point of care (at a primary care clinic, for example). The capturing of the assay result in a cell phone also facilitates documentation and archiving of test results, as well as real-time relaying of test results or aberrant test patterns to the care provider, if so desired. Such an assay could potentially be used in many different clinical settings. New-onset SLE patients without renal involvement at baseline need to be monitored frequently in order to detect onset of renal disease (which would necessitate more aggressive therapy). The onset of renal disease in SLE is silent (i.e., the patient may have non-specific proteinuria without any attendant symptoms). Currently, patients do not have access to a technology that will enable them to monitor their renal status from the comfort of their home. In this context, the use of LFA tests such as the one detailed here could potentially fill this void. Indeed, early detection of renal disease and prompt treatment have been shown to improve patient and renal outcome (43–46).

The ALCAM LFA test could also be very useful in patients already diagnosed with lupus nephritis. The natural course of the disease is marked by periods of quiescence interrupted by disease exacerbations, termed renal flares (47–52). Regular home monitoring of uALCAM or monitoring at a primary care clinic could potentially be useful in detecting an impending renal flare. Urine monitoring for disease biomarkers (such as uALCAM) at regular intervals could potentially be life-saving because up to 60% of adults and 80% of children with SLE develop LN, with 10–30% progressing to end-stage renal disease (ESRD) within 15 years of diagnosis, despite aggressive treatment. This less-expensive and easily-repeatable alternative to a repeat renal biopsy if offered as a home test, could greatly facilitate timely adjustment of immunosuppressants, without the risks of repeat renal biopsies.

Several limitations of the study warrant attention. The ALCAM LFA assay reported has only been verified and validated in a laboratory setting. A clinical trial is needed to field-test this assay platform and to ascertain its clinical feasibility and utility. Larger studies are needed to confirm if un-normalized uALCAM (AUC=0.93 by LN vs HC, AUC=0.86 by LN vs other SLE) is superior to HVEM normalized uALCAM (AUC=0.91 by LN vs HC, AUC=0.76 by LN vs other SLE) in identifying active lupus nephritis by LFAs. Also, further studies are in need to investigate the difference between diagnostic power of ELISA-derived HVEM-normalized uALCAM (AUC=0.53 by LN vs other SLE) and LFA-derived HVEM-normalized uALCAM (AUC=0.76 by LN vs other SLE). With respect to the LFA platform, both further shortening the assay time and rendering the assay compatible with undiluted urine samples will enhance the uptake of this assay among the target population. Further improvements to the presented technology would entail broadening the range of smartphones usable as readers or introducing an inexpensive standard reader, assurance of a reproducible supply of the critical antibodies, and translation to skilled manufacturing under formal quality control, and potentially the implementation of a multi-analyte panel for a greater diagnostic performance.

## Data availability statement

The original contributions presented in the study are included in the article/**Supplementary Material**. Further inquiries can be directed to the corresponding authors.

## Ethics statement

The studies involving human participants were reviewed and approved by IRB of University of Houston and John Hopkins Medical University. The patients/participants provided their written informed consent to participate in this study.

## Author contributions

Conceptualization: CM, RW, BV, KK, JB. Methodology: RL, CM, RW, SS, AD. Investigation: RL, CM, RW, BV, KK. Visualization: CM, RW, RL. Supervision: CM, RW, RL, BV, KK. Writing—original draft: RL. Writing—review & editing: RL, CM, RW, MP, SN. All authors contributed to the article and approved the submitted version.
