# Correction: A novel technology for home monitoring of lupus nephritis that tracks the pathogenic urine biomarker ALCAM

**DOI:** 10.3389/fimmu.2025.1734343

**Published:** 2025-11-21

**Authors:** Rongwei Lei, Binh Vu, Katerina Kourentzi, Sanam Soomro, Adheesha N. Danthanarayana, Jakoah Brgoch, Suma Nadimpalli, Michelle Petri, Chandra Mohan, Richard C. Willson

**Affiliations:** 1 Department of Biomedical Engineering, University of Houston, Houston, TX, United States; 2 William A. Brookshire Department of Chemical and Biomolecular Engineering, University of Houston, Houston, TX, United States; 3 Department of Chemistry, University of Houston, Houston, TX, United States; 4 Division of Rheumatology, Johns Hopkins University School of Medicine, Baltimore, MD, United States; 5 Department of Biology and Biochemistry, University of Houston, Houston, TX, United States; 6 Escuela de Medicina y Ciencias de Salud, Tecnológico de Monterrey, Monterrey, NL, Mexico

**Keywords:** lupus nephritis, nanophosphors, lateral flow assay, biomarker, diagnostic

The **Conflict of interest** statement was erroneously given as: “RW is a named inventor on IP covering the use of nanophosphors in lateral flow assays, and may receive income from its commercialization by Clip Health, Inc. The remaining authors declare that the research was conducted in the absence of any commercial or financial relationships that could be constructed as a potential conflict of interest”.

The correct **Conflict of interest** statement is “RW is a named inventor on IP covering the use of nanophosphors in lateral flow assays, and may receive income from its commercialization by Clip Health, Inc. Authors RW, KK and BV acknowledge their financial interest in Glow Nanotech which at the time was developing a different LFA technology. The remaining authors declare that the research was conducted in the absence of any commercial or financial relationships that could be constructed as a potential conflict of interest”.

The original version of this article has been updated.

