# Supplementary material for: A novel technology for home monitoring of lupus nephritis that tracks the pathogenic urine biomarker ALCAM
Source: Front Immunol. 2022 Dec 9;13:1044743. doi: 10.3389/fimmu.2022.1044743 (PMC9780296; doi:10.3389/fimmu.2022.1044743)
Supplement: Supplementary file 1 [file DataSheet_1.docx]

**A novel technology for home monitoring of lupus nephritis that tracks the pathogenic urine biomarker, ALCAM**

Rongwei Lei^1^, Binh Vu^2^, Katerina Kourentzi^2^, Sanam Soomro^1^, Adheesha N Danthanarayana^3^, Jakoah Brgoch^3^, Suma Nadimpalli^1^, Michelle Petri^4^, Chandra Mohan^1*^, Richard C Willson^2,5,6,*^

^1^Department of Biomedical Engineering, University of Houston, Houston, TX, 77004, USA. ^2^William A. Brookshire Department of Chemical and Biomolecular Engineering, University of Houston, Houston, TX, 77204, USA.

^3^Department of Chemistry, University of Houston, Houston, Texas 77204, USA

^4^Division of Rheumatology, Johns Hopkins University School of Medicine, Baltimore, MD.

^5^Department of Biology and Biochemistry, University of Houston, Houston, Texas 77204, USA.

^6^Escuela de Medicina y Ciencias de Salud, Tecnológico de Monterrey, Monterrey, Nuevo León 64710, Mexico.

**Corresponding authors**

*Drs. Mohan and Willson are co-senior authors

[cmohan@central.uh.edu](mailto:cmohan@central.uh.edu) and [willson@uh.edu](mailto:willson@uh.edu)

**1. Supplementary Methods**

**1.1 Nanophosphor preparation, milling, sieving, fractionation, and silica encapsulation** The preparation of green phosphors SrAl_2_O_4_: Eu^2+^, Dy^3+^ (SAO) has previously been described [23,24]. For initial size reduction, 10 g of green phosphors (Ultra Green V10 Glow in the Dark Powder, Glow, Inc.) was mixed in 100 ml of anhydrous ethanol and ball-milled for 21 days in a ceramic milling jar with zirconia grinding media at 110 rpm.

The dried SAO particles were then sieved through 50, 100, 150, and 200 U.S Standard Screen size sieves (Cole-Parmer® Testing Sieves) in a sieve shaker (Cole-Parmer,  # EW-59986-01

115V) for 6 hours.

SAO was further size-reduced to 200-300 nm by having particles that were retained on 150 and 200 sized sieves by differential centrifugal sedimentation (Beckman Coulter Avanti J-E centrifuge). Briefly, 50 mg particles were added to each of twelve 50-ml falcon tubes with 40 ml of ethanol. After 5 min sonication and 1 min. vortexing, tubes were centrifuged at 1004 rcf for 5 min. 25 ml of supernatant was collected as Fraction #1, and then tubes were refilled with 40 mL ethanol, and the process was repeated three times. Next, another twelve 50 ml tubes were each filled with 40 ml of Fraction #1. After 5 min sonication and 1min. vortexing, the tubes were spun at 1178 rcf for 10 min. Again, 25 ml of supernatant was removed, and the tubes were refilled with Fraction #1 until all the Fraction #1 was processed. Finally, fractionated particles in the twelve tubes were combined and stored in anhydrous ethanol. The concentration was calculated from the dried particle mass measured on an analytical balance (Model no. XS64, Mettler Toledo).

Next, fractionated SAO particles were silica-encapsulated to increase water stability. The particles were placed in a 4 °C bath and sonicated (Fisher Scientific FS30) for 15 min. Then, a mixture of 221.6 µl anhydrous ethanol, 246.7 µl DI water (Millipore Milli-Q), and 6.7 µl tetraethyl orthosilicate (TEOS; 99%; Sigma-Aldrich) was added to 2 mg/ml sonicated particles. The mixture was sonicated for another 5 min for dispersal. Then, 30 µl of aqueous ammonium hydroxide (28–30%; Sigma-Aldrich) was added, followed by another 30 min sonication and rotation for 7.5 hours at a speed of 20 rpm at room temperature. Finally, the encapsulated particles were washed three times for 3 min at 3000 rcf in anhydrous ethanol by centrifugation (Eppendorf Centrifuge 5418). The particles were finally resuspended in 1 ml anhydrous ethanol and were ready for salinization and functionalization.

**1.2 Silanization and functionalization of nanophosphors with antibodies**

Aldehydes were introduced onto the silica-encapsulated SAO particles using triethoxysilylbutyraldehyde (TESBA; Gelest, Inc., cat no. SIT8185.3; Morrisville, PA). Because TESBA is rapidly oxidized, the reagent was flushed with dry nitrogen, wrapped with parafilm after each use, stored in the desiccator, and replaced every six months. First, 1 ml of the above-mentioned silica-encapsulated phosphors in ethanol was centrifuged for 3 min at 3000 rcf, and the top 216 µl of ethanol was removed without disturbing the settled nanoparticles. A mixture of 155 µl of TEOS, 5 µL of TESBA, and 1393 µl of anhydrous ethanol was made. Next, 10 µl of the mixture was added to the particles. A separate solution of 189 µl of DI water and 16.7 µl of aqueous ammonium was prepared and added to the nanoparticles. The resulting 2 mg/ml phosphors suspension (in 1 ml) was sonicated for 10 min and then placed on the rotator at 20 rpm for 12 h at room temperature. Then the particles were washed three times for 3 min at 3000 rcf in anhydrous ethanol by centrifugation, followed by one wash with DI water and one wash with phosphate-buffered saline, pH 8 (PBS; Takara Bio) for 5 min at 6500 rcf, and finally resuspended in 800 µl PBS.

**1.3 Optimization protocol for antibody functionalization.** Twenty-five ul of 1 mg/ml of anti-ALCAM antibody (MAB6561) and 50 µl of freshly-prepared 1 M NaBH_3_CN (Chem-Impex International, Inc; Wood Dale, IL cat no. 04836) were added to 400 µl of particles in a 1.5 ml low-binding tube (Thermo Fisher). To conjugate the anti-HVEM antibody (MAB356) to the particles, 25 µl of 1 mg/ml antibody and 100 ul 1M NaBH_3_CN were added to the other tube. Tubes were filled with PBS to 500 µl, sonicated for 5 min, and rotated at room temperature at 20 rpm for 1.45 h. Particles were then washed in 1 ml PBS, pH 7.4, for 5 min at 6500 rcf, and resuspended in 112.5 µl volume in PBS, pH 7.4.

Finally, to passivate the particles, 375 µl of 54 mg/ml of bovine serum albumin (BSA; 98%; Sigma-Aldrich) in PBS, pH 7.4 and 12.5 ul of 1 M NaBH_3_CN were added to the particles. Particles were passivated on the rotator for 4 h at 20 rpm, at room temperature. After passivation, particles were washed three times for 3 min each at 3000 rcf with PBS, pH 7.4, and resuspended in 25 µl borate storage buffer (10 mM sodium borate (J.T. Baker), 150 mM NaCl (Macron), 0.1% BSA, 0.04% 40,000 ave. Mol. Wt polyvinylpyrrolidone (PVP-40; Sigma-Aldrich), 0.025% Tween-20 (Sigma-Aldrich), pH 8.5) and stored at 4 ºC.

**1.4 Assembly of LFA strips**

LFA strips consisting of a nitrocellulose membrane (FF80HP or FF120HP from Cytiva, 3.0 cm length), Whatman standard 14 sample pad (1.1 cm length), and Whatman CF 5 absorbent pad (2.2 cm length), all assembled onto an adhesive backing card (MIBA-020; DCN Diagnostics, 6.1 cm length). Mouse anti-ALCAM antibody (AF656, R&D Systems) and mouse anti-HVEM antibody (AF351, R&D, System) were reconstituted to 1 mg/ml in sterile PBS (Dulbecco's PBS; Caisson Labs) for the test line. Goat anti-mouse IgG (ABGAM-0500; Arista) was diluted in sterile PBS for the control line, to the concentration stated in the LFA strip preparation protocol in the main text. Antibodies were dispensed onto the nitrocellulose membrane using a BioDot dispenser (XYZ30600124) at a rate of 1 µl/cm. The assembly was then dried at 37 ºC for 30 min in an incubator (Robbins Scientific Micro Hybridization Incubator 2000) and then cut into 3 mm wide strips using a ZQ2000 Guillotine Cutter. Strips were stored in 50 ml conical screw cap centrifuge tubes with one bag of silica gel desiccant (S-3905; Uline) at room temperature for at least one day before use.

**1.5 FluorChem-based and Smartphone-based imaging of nanophosphors**

LFA strips were imaged on a FluorChem gel documentation system (Alpha Innotech Corp., San Leandro, CA), with an exposure time of 4 s and pixel binning of 4 [24]. LFA strips were also imaged with a smartphone-based imaging platform composed of an iPhone 5S and a 3D-printed attachment. An app generously provided by Luminostics, Inc was used to control the rear camera and flash. The flash excites the nanoparticles for 3 s, and 0.5 s exposure time image acquisition occurs after a 100 ms time delay. Four images are acquired and averaged to reduce background noise and increase reproducibility [24]. The reusable 3D printed iPhone 8 attachment prototype (<$10) was designed to position the LFA test cartridge (part number MICA-125; DCN Diagnostics) and to easily slide onto a smartphone like a protective case. This inexpensive plastic attachment provides darkness and a clear window for imaging.

**1.6 Macro**

The image J measurements setup:

run("Line Width... ");

run("Set Scale...", "distance=1 known=1 pixel=1 unit=unit");

run("Profile Plot Options...", "width=450 height=200 minimum=0 maximum=0 interpolate");

run("Set Measurements...", "area redirect=None decimal=4");

run("Colors...", "foreground=black background=black selection=yellow");

run("Close");

**Supplementary** Table S1: Demographic and clinical characteristic of validation study cohort.

| Variable | ALN  n=30 | ANR  n=18 | Inactive  n=29 | HC  n=30 |
| --- | --- | --- | --- | --- |
| Race | | | |  |
| White | 7 | 4 | 12 | 0 |
| Black | 20 | 14 | 15 | 0 |
| Hispanic | 0 | 0 | 0 | 0 |
| Asian | 2 | 0 | 1 | 0 |
| Other | 1 | 0 | 1 | 0 |
| Age (yr) | | | |  |
| Mean | 32±9.4 | 41±12.1 | 48±13.0 | 0 |
| Range | 21-56 | 22-62 | 23-74 | 0 |
| Sex | | | |  |
| Female | 26 | 18 | 26 | 0 |
| Male | 4 | 0 | 3 | 0 |
| SLEDAI | 8±4.6 | 6±2.8 | 1±1.0 | 0 |
| rSLEDAI | 5.3±4.2 | 0 | 0 | 0 |

HC: healthy control; Inactive: inactive SLE patients; ANR: active non-renal SLE patients; ALN: active renal lupus nephritis patients; SLEDAI: Systemic Lupus Erythematosus Disease Activity Index; rSLEDAI: renal SLEDAI

**Supplementary** Table S2. Commercial Anti-ALCAM antibodies tested for inclusion in the LFA

| Anti-ALCAM antibodies | Vendor | Cat No. | Host | Concentration spotted on membrane |
| --- | --- | --- | --- | --- |
| mAb-1A | R&D Systems | MAB6561 | Mouse | 1 mg/ml |
| mAb-2A | BioLegend | 343902 | Mouse | 0.5 mg/ml |
| mAb-3A | Thermo Fisher Scientific | MA515421 | Mouse | 1 mg/ml |
| mAb-4A | BD | 559260 | Mouse | 0.5 mg/ml |
| mAb-5A | Santa Cruz | Sc-74558 | Mouse | 0.2 mg/ml |
| pAb-1A | R&D Systems | AF656 | Goat | 1 mg/ml |
| pAb-2A | ABclonal | A2218 | Rabbit | 1 mg/ml |
| pAb-3A | Thermo Fisher Scientific | PA5-12513 | Rabbit | 1 mg/ml |

**Supplementary** Table S3. Anti-HVEM antibodies tested

| Anti-HVEM  antibodies | Vendor | Cat No. | Host | Concentration spotted on membrane |
| --- | --- | --- | --- | --- |
| mAb-1H | R&D Systems | MAB356 | Mouse | 1 mg/ml |
| mAb-2H | BioLegend | 318802 | Mouse | 0.5 mg/ml |
| pAb-1H | R&D Systems | AF351 | Goat | 1 mg/ml |
| pAb-2H | Thermo Fisher Scientific | PA518451 | Goat | 0.5 mg/ml |

**Supplementary** Table S4. Control line antibodies

| Control line antibody | Vendor | Cat No. | Host | Concentration in stock |
| --- | --- | --- | --- | --- |
| pAb-anti-goat | Arista | ABDGG-0500 | Donkey | 1 mg/ml |
| pAb-anti-mouse | Arista | ABGAM-0500 | Goat | 0.5mg/ml |
| pAb-anti-rabbit | ABclonal | AS070 | Goat | 1mg/ml |

Anti-ALCAM antibodies and anti-HVEM antibodies used for initial LFA-based antibody screening. mAb, monoclonal antibody; pAb, polyclonal antibody

###

**Supplementary Figure S1: Verification of HVEM LFA assay reproducibility and stability**. A) Three different batches of anti-HVEM (pAb-1H as capture antibody) dispensed strips were assembled. One batch of anti-HVEM (mAb-1H as detection antibody) conjugated nanophosphors was mixed with spiked running buffer-1A and loaded onto three batches of HVEM LFA strips. No significant differences were found in linearity and LoD (0.5 ng/ml) among the three standard curves. B) Assessing the reproducibility of anti-HVEM conjugated nanophosphors preparation. One batch of anti-HVEM dispensed strips was prepared. Three batches of conjugated anti-HVEM nanophosphors were individually mixed with spiked running buffer-1A and loaded onto the HVEM LFA strips. No significant differences were found in linearity and LoD (0.5 ng/ml) among the three batches of conjugated anti-HVEM nanophosphors. C) Stability of anti-HVEM conjugated nanophosphors. Anti-HVEM conjugated nanophosphors were freeze-dried and then reconstituted one day, one month, two months, and three months after storage, respectively. At each time point, the reconstituted nanophosphors were mixed with running buffer-II and loaded onto HVEM LFA to construct standard curves. No significant differences were found in linearity and LoD (0.5 ng/ml) after different storage durations, verifying the stability of nanophosphors at room temperature.


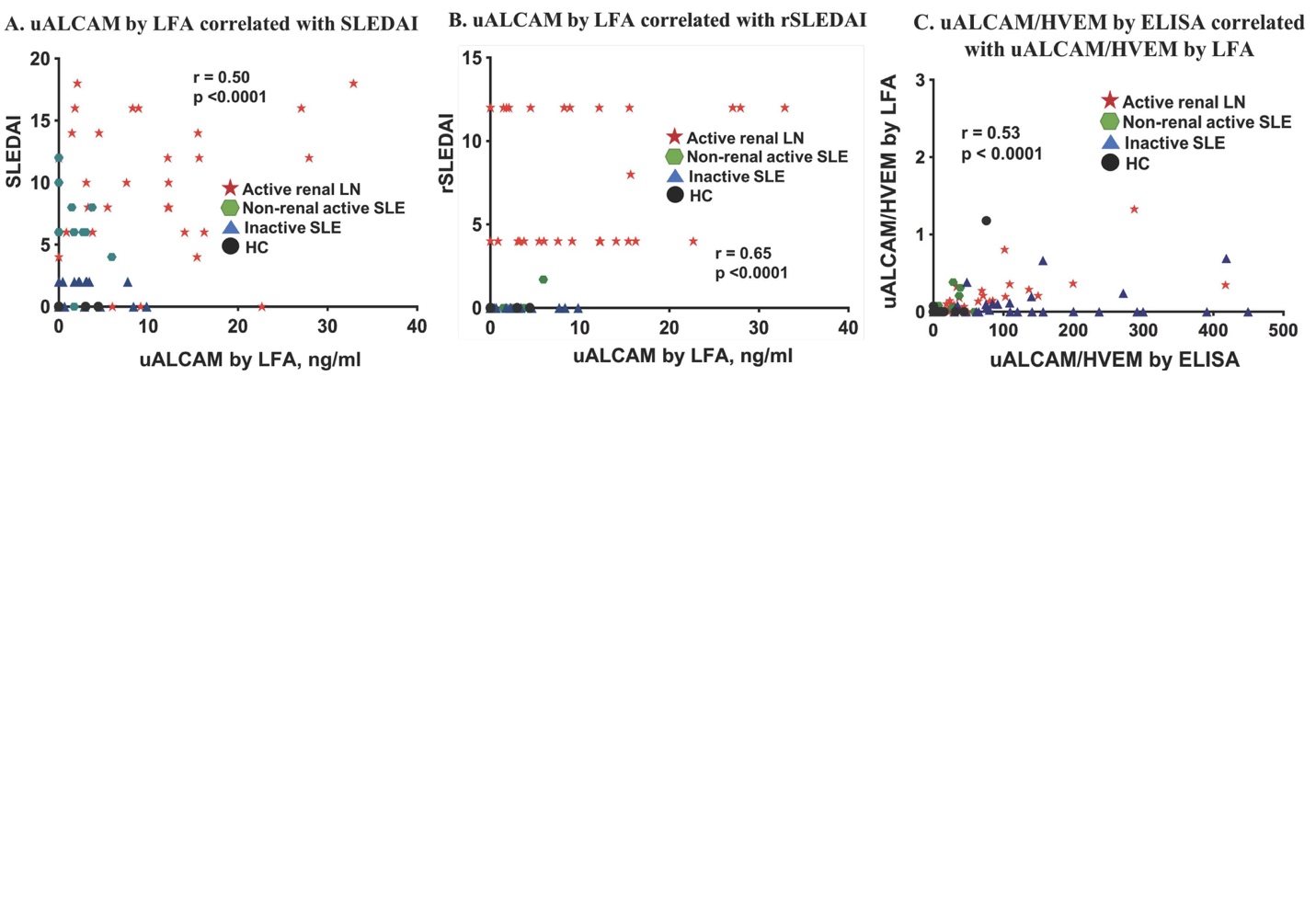


### Supplementary Figure S2: the diagnostic profile of lateral flow assay and ELISA in clinical samples. In four groups of subjects (30 HC, 29 inactive, 18 ANR, and 30 ALN), uALCAM and uHVEM were assayed by ELISA and LFA, separately. uALCAM as assayed using the LFAs was significantly (Spearman) correlated with the disease activity metrics, SLEDAI (A) and rSLEDAI (B). C) ELISA-derived HVEM-normalized uALCAM as assayed by ELISA was significantly (Spearman) correlated with LFA-derived HVEM-normalized uALCAM as assayed by LFA. *p < 0.05, **p < 0.01, ***p < 0.001, ****p < 0.0001. HC: healthy control; inactive: inactive SLE patients; ANR: active non-renal SLE patients; ALN: active renal lupus nephritis patients; (r)SLEDAI: (renal) SLE Disease Activity Index (SLEDAI)


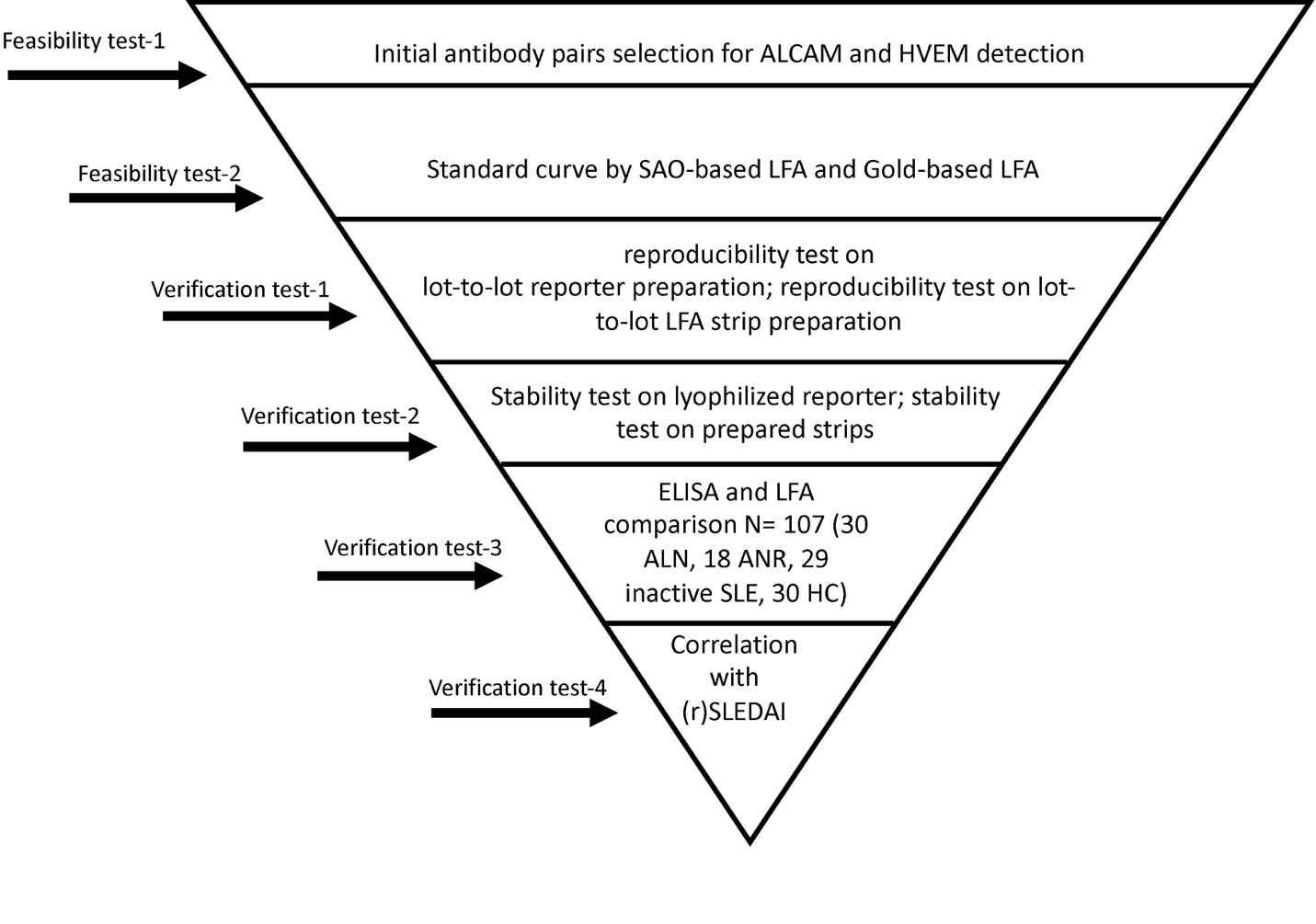


Supplementary Figure S3: An overview of the current study to develop a nanophosphors-based lateral flow assay for lupus nephritis flare biomarker (ALCAM) and urine normalizer (HVEM) detection. Initially, antibody pairs were comprehensively interrogated for the optimal lateral flow assay performance. Antibody pairs with the highest brightness and signal over noise ratio were selected. Based on the antibody pairs, nanophosphors-based lateral flow assay and traditional gold nanoparticles-based lateral flow assay were individually evaluated by building serial dilution. To verify the strength of the nanophosphors-based lateral flow assay, comprehensive reproducibility and stability of nanophosphors and strips were evaluated by demonstrating serial dilutions. To further verify the diagnostics power of the index test, 107 urine samples were individually assayed by lateral flow assay for uALCAM and uHVEM levels, as well as by reference test-ELISA for uALCAM and uHVEM levels. The correlation of uALCAM assayed by LFA to uALCAM assay by ELISA, of uALCAM assayed by LFA to (r)SLEDAI were demonstrated, likewise the HVEM. The diagnostic power of uALCAM and uALCAM corrected by uHVEM assayed by LFA was also demonstrated in ALN vs. HC and ALN vs. other lupus.
